# A comprehensive metagenomics framework to characterize organisms relevant for planetary protection

**DOI:** 10.1186/s40168-021-01020-1

**Published:** 2021-04-01

**Authors:** David C. Danko, Maria A. Sierra, James N. Benardini, Lisa Guan, Jason M. Wood, Nitin Singh, Arman Seuylemezian, Daniel J. Butler, Krista Ryon, Katerina Kuchin, Dmitry Meleshko, Chandrima Bhattacharya, Kasthuri J. Venkateswaran, Christopher E. Mason

**Affiliations:** 1 Department of Physiology and Biophysics, Weill Cornell Medicine, New York, NY 10065 USA; 2 Tri-Institutional Computational Biology & Medicine Program, Weill Cornell Medicine, New York, NY USA; 3 The HRH Prince Alwaleed Bin Talal Bin Abdulaziz Alsaud Institute for Computational Biomedicine, Weill Cornell Medicine, New York, NY 10065 USA; 4 Biotechnology and Planetary Protection Group, Jet Propulsion Laboratory, Pasadena, CA 91109 USA; 5 WorldQuant Initiative for Quantitative Prediction, Weill Cornell Medicine, New York, NY USA; 6 The Feil Family Brain and Mind Research Institute, Weill Cornell Medicine, New York, NY USA

**Keywords:** Planetary Protection, Spacecraft Assembly Facility, Extremophile, Microbial profiling

## Abstract

**Background:**

Clean rooms of the Space Assembly Facility (SAF) at the Jet Propulsion Laboratory (JPL) at NASA are the final step of spacecraft cleaning and assembly before launching into space. Clean rooms have stringent methods of air-filtration and cleaning to minimize microbial contamination for exoplanetary research and minimize the risk of human pathogens, but they are not sterile. Clean rooms make a selective environment for microorganisms that tolerate such cleaning methods. Previous studies have attempted to characterize the microbial cargo through sequencing and culture-dependent protocols. However, there is not a standardized metagenomic workflow nor analysis pipeline for spaceflight hardware cleanroom samples to identify microbial contamination. Additionally, current identification methods fail to characterize and profile the risk of low-abundance microorganisms.

**Results:**

A comprehensive metagenomic framework to characterize microorganisms relevant for planetary protection in multiple cleanroom classifications (from ISO-5 to ISO-8.5) and sample types (surface, filters, and debris collected via vacuum devices) was developed. Fifty-one metagenomic samples from SAF clean rooms were sequenced and analyzed to identify microbes that could potentially survive spaceflight based on their microbial features and whether the microbes expressed any metabolic activity or growth. Additionally, an auxiliary testing was performed to determine the repeatability of our techniques and validate our analyses. We find evidence that JPL clean rooms carry microbes with attributes that may be problematic in space missions for their documented ability to withstand extreme conditions, such as psychrophilia and ability to form biofilms, spore-forming capacity, radiation resistance, and desiccation resistance. Samples from ISO-5 standard had lower microbial diversity than those conforming to ISO-6 or higher filters but still carried a measurable microbial load.

**Conclusions:**

Although the extensive cleaning processes limit the number of microbes capable of withstanding clean room condition, it is important to quantify thresholds and detect organisms that can inform ongoing Planetary Protection goals, provide a biological baseline for assembly facilities, and guide future mission planning.

Video Abstract

**Supplementary Information:**

The online version contains supplementary material available at 10.1186/s40168-021-01020-1.

## Background

With the increasing number of spaceflights, microbial colonization of SpacecraftsAssembly Facilities (SAF) surfaces is a major concern [1]. Planetary protection research efforts at the Jet Propulsion Laboratory (JPL) at NASA seek to develop technologies for cleaning and sterilization of spacecraft prior to launch to reduce any terrestrial microbial contamination [2]. Clean rooms of the SAFs are the final step before spacecraft launch into space. SAFs are specialized to minimize both the influx and residence time of particulate matter via stringent methods of air-filtration and cleaning [3]. Particulate matter includes dust that workers might bring with them, such as fabric lint or dead skin, as well as microbes and biological entities. The clean room ventilation system circulates air through HEPA filters specially designed to last several decades [4, 5].

As such, SAF environments are highly selective for microorganisms that can tolerate unique and repeated cleaning conditions, such as chemical oxidizing agents, desiccation, and UV irradiation [6, 7]. Microorganisms recovered using culture techniques on SAF surfaces have been characterized as species of Archaea, Bacteria, and Fungi that are commonly associated with human commensals, but also some which are found in soil, airborne dust, and urban environments [1, 8, 9]. Studies have repeatedly shown species of the extremely hardy, anaerobic, and spore-forming kind are the most highly represented in samples collected from vacuum devices and facility surfaces, including fungal genera such as *Alternaria*, *Aspergillus*, *Bipolaris*, *Candida*, *Cladosporium*, *Fusarium*, *Mucor*, *Penicillium*, and *Trichoderma*; and bacterial genera *Acinetobacter*, *Alcaligenes*, *Bacillus*, *Propionibacterium*, *Corynebacterium*, *Pantoea*, *Brevibacterium*, *Flavobacterium*, *Micrococcus*, *Staphylococcus*, and *Streptococcus* [9–11]. However, current identification methods are insufficient for detecting low-abundance microorganisms, as has been studied in SAFs and other environments [12–14]. Accordingly, improved methods and quantitative testing of microbial loads would help guide optimized methods for Planetary Protection (PP) protocols in SAFs and for spacecraft construction.

Implementation of reliable planetary protection (PP) protocols will not only ensure that extraterrestrial bodies can remain biological preserves for scientific investigations, but also minimize the risk of human exposure to contaminants [15], especially in long-term missions and the effects it may have on the human health and microbiome [16]. Unlike ordinary environmental or clinical samples, clean-room samples often have very little DNA, due to the nature of extensive cleaning and process control of surfaces, air, and particulate matter. Clean-room samples should also contain very little human DNA, few unique reads overall (as they are sequenced to saturation), higher polymerase chain reaction (PCR) duplicates, and low overall complexity. By establishing a baseline for these metrics, we aimed to develop a comprehensive profile of species present in clean rooms, even at low abundances, and quantify the risk and detect contamination incidents in SAFs.

For this purpose, we processed and analyzed 51 samples from the clean rooms of the SAF at JPL. Samples correspond to SAF surfaces, including wipe solution of filters and particles collected via vacuum devices resuspended in particle solution. In order to establish and evaluate a dedicated cleaning plan, we first quantify the richness of microbial communities and their taxonomic composition. We then identify the potential risk of microbes based on the microbe viability by annotating the microbe functional features, including the potential radiation resistance, biocide resistance, presence of extremophile features, and genes associated that could help microbes survive outside Earth. Furthermore, we evaluate the rate of growth for major taxa in clean rooms, providing an estimate of the risk.

## Methods

### Samples collection and processing

A total of 51 samples were collected and prepared by JPL and sent to Weill Cornell Medicine (WCM) for processing. Samples were obtained from controlled cleanroom environments ranging from certified ISO-5 (Class 100) to ISO-8.5 (Class 300,000) and stored at 4 °C until processed. In cleanrooms, an all-purpose cleaning and degreasing agent (Kleenol 30, Accurate Industrial Supply, Inc., Cerritos, CA, USA, Cat #: J-CC-00040) was used to maintain cleanliness of the floor. Surface cleaning procedures were performed twice a day in the cleanroom during periods when spacecraft componentry was actively undergoing assembly.

For each sample type, 20–40 mL of solution was sent to WCM for extraction and analysis. Samples that were extracted at JPL were stored at − 20°C before sending to WCM. Sample types were spanned in five sample categories described as follows:

- Category 1 (Cat. 1) DNA Samples: A subset of sample solutions from facility surface wipe samples and flow bench pre-filters were processed for DNA extraction at JPL. Sample solutions obtained from the previously described methods were filter concentrated (MilliporeSigma, 50kD Amicon® Ultra 15 mL Centrifugal Filters) to a final volume of 250 µL. To the concentrated samples, 200 µL of lysis buffer (Qiagen, Buffer ATL, UCP Pathogen Mini Kit) was added and incubated at 56 °C with continuous shaking at 600 rpm for 10 min. The lysed solution was added to bead beating tubes (OPS Diagnostics, 100 mm/400 mm Acid-Washed Silica Beads) and loaded onto a vortex mixer at max speed for 10 min ± 30 s. The tubes were centrifuged for 2 min at 13,000×*g*. Final lysate was aspirated from the tubes and loaded onto the QIAcube automated DNA extraction instrument (Qiagen) with a Qiagen UCP Pathogen Mini Kit protocol following manufacturer specifications for microbial samples.
- Category 2 (Cat. 2) Facility Surface Wipe Samples: Wipe samples were collected from the floor, walls, and work surfaces of multiple cleanrooms that process biologically sensitive spaceflight hardware. Samples were taken at various time points based on access availability. Samples were collected using sterile polyester wipes (Texwipe Co., TX3211 SterileWipe) pre-moistened with water (ThermoFisher Scientific, UltraPure™ DNase/RNase-Free Distilled Water). Post-sample collection, wipes were placed back into the 50-mL polystyrene tube (BD Falcon, 50 mL Conical Tube) for ease of transporting back to the laboratory. Then, wipes were individually transferred to 500 mL polystyrene storage bottles (Corning) and 100 mL of dissociation buffer (10 mM Tris, 1 mM EDTA, pH 8.0, 0.05% Tween 80 [v/v]) was added to each bottle. Bottles were placed in an ultrasonic bath containing 0.05% Tween (v/v) and sonicated for 5 min ± 15 s at 25 kHz. Bottles were then shaken for 30 min ± 1 min at 200 rpm on an orbital shaker. Wipe samples that were expected to be ultra-low in biomass based on engineering judgement and preliminary testing, (i.e., ISO-5 cleanroom environments, aseptic-like processing facilities), several wipe solutions were combined together and filter concentrated (MilliporeSigma, 50 kD Amicon® Ultra 15 mL Centrifugal Filters). A control wipe solution which followed the same procedures as surface wipe samples but did not come into contact with any facility surfaces was sent as a background control.
- Category 3 (Cat. 3) Filter Solution: Pre-filter samples were collected from ISO-5 flow benches within JPL cleanroom environments at the time of flow bench recertification. These ISO-5 flow benches and associated filters were only utilized for cleanroom use. A forensic vacuum (3M, Trace Evidence Vacuum A-6510) was used to collect particulates from off the pre-filter. Pre-sealed vacuum filters (3M, Trace Evidence Vacuum Filters A-6512, 97% retention rate for 0.1-micron particles) were attached to the forensic vacuum and used to vacuum the cleanroom-facing side of the flow bench pre-filters. Vacuum filters were removed from the pre-sealed filter unit and individually transferred to polystyrene 500 ml storage bottles (Corning, Corning, NY). A total of 50 mL of dissociation buffer (10 mM Tris, 1 mM EDTA, pH 8.0, 0.05% Tween 80 [v/v]) was added to each bottle. Bottles were placed in an ultrasonic bath containing 0.05% Tween (v/v) and sonicated for 5 min ± 15 s at 25 kHz. Bottles were then shaken for 30 min ± 1 min at 200 rpm on an orbital shaker. Control filter solutions for each type of flow bench pre-filter were obtained from unused flow bench pre-filters and sent as background controls.
- Category 4 (Cat. 4) Vacuum Particle Solution: Cleanroom vacuum samples were collected from the dust bags of certified cleanroom vacuums (TBD Model) from various JPL cleanrooms. These vacuum dust bag samples were chosen to represent the highest concentration of biomass obtained from a cleanroom environment. Particles from the dust bag were transferred to a polystyrene 500 mL storage bottle (Corning, Corning, NY). A total of 75 mL of dissociation buffer (10 mM Tris, 1 mM EDTA, pH 8.0, 0.05% Tween 80 [v/v]) was added to each bottle. Bottles were placed in an ultrasonic bath containing 0.05% Tween (v/v) and sonicated for 5 ± 15 s min at 25 kHz. Bottles were then shaken for 30 min ± 1 min at 200 rpm on an orbital shaker. Dissociation buffer without any added sample was used as a background control.
- Category 5 (Cat. 5) DNA Replicate Samples: Five 10 µL mL aliquots from one DNA sample obtained from the Spacecraft Assembly Facility (ISO-7) at JPL were sent to WCM to evaluate any variability within one sample that may have arisen from processing, sequencing, or analysis. This sample was prepared given the methods detailed above and selected for replicate sequencing based on its above-detection levels of DNA as quantified by Qubit (ThermoFisher, Qubit dsDNA HS Assay Kit). Control DNA from a sample that did not come into contact with any facility surfaces was sent as a background control.

### DNA extraction

The study plan included 51 total NASA samples (including 9 controls from JPL) and 3 negative controls from Weill Cornell Medicine (*n* = 54 total), and all were subjected to quality filtering (QC) (Table S1). Two libraries failed QC thresholds (sample 2-11 and sample 5-4). The remaining samples were sequenced and analyzed.

Preparation of samples was adapted from the Maxwell RSC Blood DNA protocol (Promega AS1400) on the Maxwell RSC 48 Machine. The samples were first vortexed to homogenize. Maxwell cartridges were prepared by placing 300 μL of lysis buffer in well 1, plunger in well 8, and an elution tube with 50 μL elution buffer into the tube holder. A minimum of 10 μL and maximum of 100 μL per sample, depending on availability, was transferred to Maxwell Cartridge well using a sterile DNA/RNA and DNAse/RNAse free pipette tip. Contamination was minimized by spraying down each deck tray with ethanol and allowing it to dry, followed by a UV sterilization. Elution tubes were immediately sealed after ≈36 min and DNA was transferred to an Eppendorf 96 well PCR plate (cat. 4095-2320).

### Library preparation and sequencing

The extracted DNA was taken through the Nextera Flex protocol by Illumina. Briefly, 10 μL of extracted DNA was taken into library prep protocol. DNA was bound to beads and tagmented using transposase technology (Illumina DNA prep. protocol Document 1000000025416 v09). 96 plex Nextera Illumina Combinatorial Dual indexes (CD) were added to uniquely barcode each library. Libraries were amplified with a 12-step PCR reaction, following Illumina guidelines for the lowest input. Libraries were cleaned up with a left-sided size selection using provided sample preparation beads from Beckman Coulter. A bead ratio of 0.9x to sample was used for this size selection. The right-sided size selection was omitted.

Libraries were then quantified using an Invitrogen Qubit Fluorometer and an Advanced Analytical/Agilent Fragment Analyzer. Next, libraries were pooled by standardized molarity calculated through average fragment size and ng/μL concentration. Many libraries were unquantifiable due to an exceptionally low input DNA, reading below the threshold of detection on the Qubit. All attempts were made to standardize molarities to reasonable concentrations (2 nM), but a high variability remained in yield going forward into sequencing.

Libraries were pooled at 2 nM and sequenced on an Illumina HiSeq 4000 in PE150 mode at the Weill Cornell Genomics Core. FASTQs were generated using Illumina basespace.

### Quality control

Adapters and low-quality bases were removed using AdapterRemoval v2 [17]. Bases with a quality of 1 were removed as were considered ambiguous bases. Reads shorter than 50 bp after trimming were also discarded. The remaining reads were aligned against the human genome with alternate contigs using Bowtie2 [18], with sensitive settings.

We used Jellyfish [19] to count k-mers on clean reads, including singletons. We calculated various statistics on k-mers using a previously-validated script [20]. Two statistics were used: (1) The fraction of k-mers which are singletons, the number of k-mers which only occurred once vs. the total number of unique k-mers; (2) k-mer entropy, the Shannon entropy calculated over the probability of drawing each k-mer at random. We counted the number of reads in each sample using standard GNU utilities.

### Taxonomic identification

Taxonomic profiles were generated by processing non-human reads with KrakenUniq (v0.3.2) [21], using a reference database based on draft and reference genomes in RefSeq Microbial [22], for Bacteria, Fungi, Virus and Archaea, ca. March 2017. KrakenUniq reports the number of unique marker k-mers assigned to each taxon, as well as the total number of reads, the fraction of available marker k-mers found, and the mean copy number of those k-mers. KrakenUniq was selected because it is highly performant, as demonstrated to have higher sensitivity than the other bioinformatic tools [23], such as MetaFlow [24] or MetaPhlAn2 [25]. After identifying taxa, we generated downstream quality control metrics for prominent microorganisms and provided an estimate of the relative abundance of taxa in each sample. We filtered taxonomic assignments to include only taxa that had at least 256 reads, 1024 unique marker k-mers, and an average minimum of 2.5 unique marker k-mer per read.

Based on our experience with environmental microbiomes, we did not automatically remove taxa found in control samples. It is difficult to distinguish taxa living in the built-environment, from closely related taxa living in the built-environment of a laboratory. Since these taxa may be ecologically important, we simply present them alongside control samples. Instead, we compared the ratio of unique markers and total number of reads assigned to a taxon between cases and controls. If a taxon had 5x as many unique markers and 2x as many reads in at least two samples compared to the most seen in any control sample that taxa was kept otherwise it was filtered. This led to the removal of 14 out of 24 taxa identified in the control samples.

### Assembly of microbial genomes

#### Metagenome-assembled genomes (MAGs)

Bacterial genomic sequences were assembled using MetaSPAdes [26] and MegaHIT [27], both state-of-the-art metagenomic assemblers using default settings. Resulting assembled sequences were binned using MetaBAT2 [28] with a minimum contig size of 1500 bp. Assembly bins were quality controlled and deduplicated using dRep [29], and the quality of the final genome set was evaluated using CheckM [30]. Only metagenome-assembled genomes (MAGs) with 80% completeness and less than 5% contamination were included. Genomes were given taxonomic classifications using GTDB-Tk [31].

#### Characterization of MAGs

After identifying MAGs, we characterized each genome for its ability to survive harsh environments. We found and annotated genes on MAGs using Prodigal [32] and PROKKA [33]. Proteins associated with the following 5 categories were evaluated: DNA repair, chemotaxis, biocide resistance, sporulation, and antimicrobial resistance, using lists present in The Microbe Directory [34]. All categories are related to significant modes of microbial resistance against the extensive sanitization and other adverse conditions of the clean rooms environments.

Many contigs, particularly larger contigs, did not precisely match any known taxa and may be from novel microbial species. In these cases, we have listed the genus that the microbe was categorized into.

### Growth rate analysis (GRA)

We performed a growth rate analysis (GRA) on all MAGs as well as on reference genomes for prominent microorganisms. GRA allows quantification of bacterial viability and activity. GRA works by identifying what fraction of bacteria are actively replicating in a sample. This can be determined from DNA because bacteria replicate their genomes from a specific, consistent, origin of replication [35]. The ratio between the number of short reads which map to the origin of replication and the opposite point of the bacterial chromosome determines a growth rate score. Scores over 1 indicate that a bacteria is replicating, with higher scores indicating a faster replication. We estimated the GRA for the two major taxa identified using our MAGs assemblies and GRiD [36].

GRiD measures the growth rate of uncharacterized bacteria or bacteria with low coverage samples and low-quality genome assemblies [37, 38]. We filtered all GRiD estimates with strain heterogeneity above 0.5 as these estimates are likely to be inaccurate.

## Results

To catalog microbes present in clean rooms from the JPL-SAF, the study plan included 51 total NASA samples (including 8 controls from JPL) Table 1 and 3 negative controls from Weill Cornell Medicine (*n* = 54 total), and all were subjected to quality filtering (QC) (Table S1). Two libraries failed QC thresholds (sample 2-11 and sample 5-4). The different sample types were classified into five categories.

**Table 1:** Number of samples in each category. Cat 1. Corresponds to DNA extracted in the clean room facilities; Cat 2. Wipe solution from surfaces; Cat 3. Filter solution from HEPA filters; Cat 4. Vacuum particle solution from ULPA filters and Cat. 5. DNA repeatability samples

Cat. #	Category	JPL-SAF*	ISO-5	Control	Total # of samples
1	Extracted DNA (JPL)	5	5	2	12
2	Wipe solution (JPL)	7	3	2	12
3	Filter solution (HEPA filters)	2	6	2	10
4	Vacuum particle solution (ULPA filters)	5	5	0	10
5	Repeatability (DNA from one JPL-SAF sample)	5	-	0	5
-	Buffer controls	-	-	2	2
Total # of samples for shotgun sequencing					51

*JPL-SAF: ISO-6 to ISO-8.5 facilities

### Quality control and identification of possible contaminants

We first checked the quality of our samples by establishing distributions of four important QC metrics. This included (1) the number of reads assigned to a species, (2) the number of unique marker k-mers assigned to a species, (3) the fraction of marker k-mers identified for a species out of all marker k-mers for that species, and (4) the ratio of reads to markers k-mers. We found that samples generally had consistent distributions of these metrics regardless of the category while distributions for control samples were more variable (Fig. S2). Based on this result we elected not to discard any samples as low quality.

One of the main challenges in metagenomics is the taxonomic identification of microorganisms due to the different approaches used by the bioinformatic tools available [23, 39]. While many algorithms and ensemble approaches exist, no single solution has the specificity to cover all cases and can claim to be wholly accurate in terms of classification accuracy. For metagenomic samples collected from the clean room, this problem is far more complex due to the low microbial cargo. In our approach, we have considered several factors for taxonomic assignment to account for such complexity. This includes to analyze the total number of reads assigned to a species, the number of unique markers k-mers used to identify a species, the ratio of reads to markers k-mers, and the identification of a species in a control. We have chosen thresholds we believe to be an effective compromise between specificity and sensitivity as a first pass at quality control, (the effects of these parameters are detailed on Fig. S6). After filtering, we identified 24 species in our controls, all of which were found in at least one “case” sample from the SAF. To determine if a species found in a control should be retained or discarded as a contaminant, we compared the ratio of reads and markers in our cases to controls (Fig. S3) and discarded species which did not consistently have stronger identifications in case samples than in controls.

We elected not to remove any taxa based on the measured relative abundance of that taxa. We chose to do this because there was no clear inflection in the distribution of taxonomic abundances that could have served as a natural threshold (Fig. S1). With no natural threshold, we elected to keep all taxonomic assignments regardless of relative abundance (provided read count minimums were met). After taxonomic filtering, we compared our QC metrics to the measures of taxonomic diversity. In general, these metrics correlated, which suggests that our taxonomic assignments corresponded to real underlying variation in our observed sequences (Fig. S4). Pearson’s correlation coefficients showed species entropy and k-mer entropy with rho = 0.625; species entropy and singleton k-mer fraction, rho = 0.499; species entropy and read count, rho = 0.781; species richness (rarefied to 1000 reads) and read count, rho = 0.886. A rarefaction analysis of our taxonomic profiles further suggested that sampled taxonomic diversity was representative of the true underlying diversity (Fig. S5).

### Taxonomic diversity is strongly associated with clean room type

**Fig. 1:**
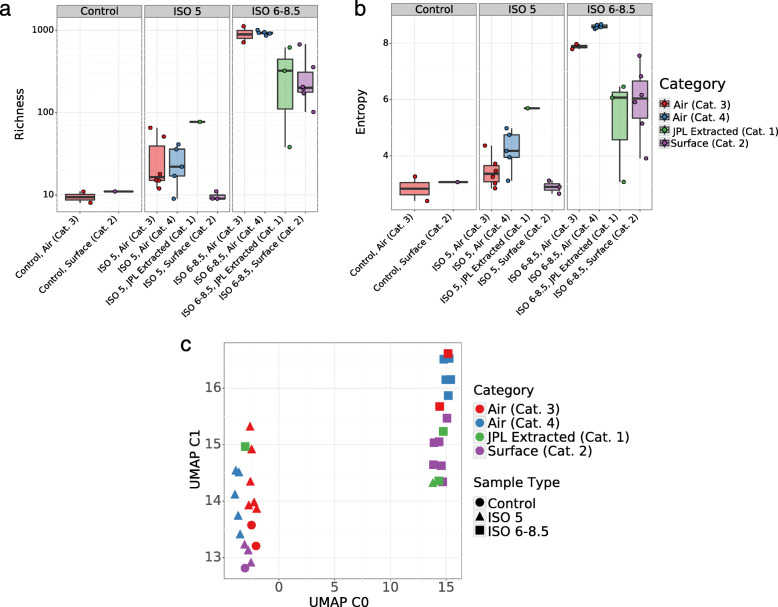
Sample Diversity. **a** Species level richness, the total number of detected species. **b** Shannon’s entropy of species abundance, a measure of alpha diversity which accounts for the relative abundance of different taxa. **c** UMAP of binary (presence/absence) taxonomic profiles

We next assessed alpha (intra-sample) and beta (inter-sample) taxonomic diversity of our samples. We found that samples from ISO-5 clean rooms were different from higher ISO categories (Fig. 1a, b), with lower diversity. Samples from ISO-5 clean rooms had lower species richness (after rarefying all samples to 1000 reads) than samples from ISO-6–8.5 facilities (*t* test, *p* = 1.17e−06) as well as entropy (*t* test, *p* = 9.71e−07). The same ISO-5 samples had higher species richness than controls (*t* test, *p* = 0.0045) as well as entropy (*t* test, *p* = 0.029). Surprisingly, this separation was restricted to ISO-5 samples. ISO-6 samples had similar alpha diversity to ISO-8.5 samples and the diversity of these samples was comparable to taxonomic diversity reported in a study of microbes in the urban environment [40].

Samples taken from HEPA and ULPA in ISO-6–8.5 clean rooms appeared to have higher diversity than samples taken from SAF surfaces, but this trend was not clear for samples from ISO-5 clean rooms. Control air and surface samples had slightly lower taxonomic diversity on average than ISO-5 samples and much lower diversity than ISO-6–8.5 samples. Analysis of beta-diversity (Fig. 1c) based on the presence and absence of taxa reduced using UMAP with Jaccard distance [41–44] showed clear differentiation between ISO-6–8.5 samples and ISO-5 samples and controls with smaller distinctions between air and surface samples.

We note that taxonomic diversity in general is not equivalent to total biomass; it is possible to have high biomass samples with low diversity and low biomass samples with high diversity. With that caveat, it seems likely that we are capturing a representative fraction of the total diversity in clean room samples. We performed a rarefaction analysis on samples from different categories in ISO-5 and ISO-6–8.5 clean rooms and found that the rarefaction reached apparent maximums (Fig. S5). This suggests that additional sampling is unlikely to identify many new taxa.

**Fig. 2:**
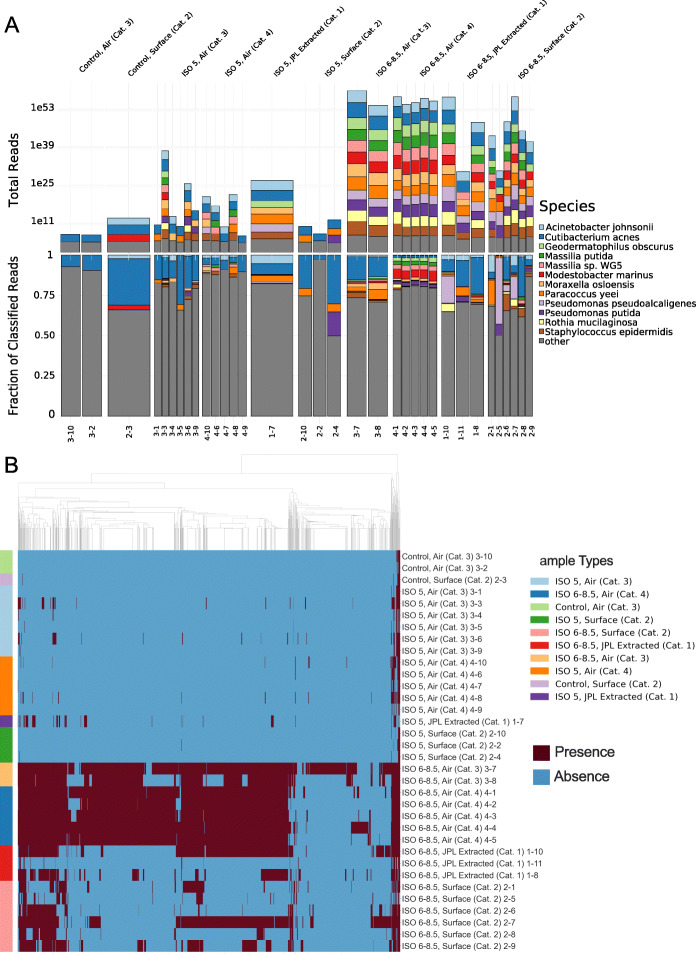
Taxonomy Classification. **a** Total number of reads assigned to each taxa (top) and proportions of each taxa (bottom) grouped by category and ISO level. **b** Heatmap of taxonomic prevalence based on presence (red) and absence (blue) for each sample

The total number of assigned taxa varied among ISO levels and categories. In general, samples from ISO-6–8.5 had more total reads than ISO-5 and control samples Fig. 2a. However, taxa such as *Acinetobacter johnsonii*, *Cutibacterium acnes*, *Moraxella osloensis*, and *Staphylococcus epidermidis* were present in both ISO-6–8.5 and ISO-5. Except for *C. acnes,* which was found in all samples, these three species were all more prevalent in ISO-6–8.5 (93–100% prevalence) than in ISO-5 samples (40–53% prevalence). Within ISO-6–8.5 there is a higher diversity of identified taxa and total reads in categories 3 and 4 (Cat. 3 and Cat. 4), then categories 1 and 2 (Cat. 1 and Cat. 2) (Fig. 2b).

While Cat. 3 and Cat. 4 correspond to air samples and Cat.1 and Cat. 2 correspond to surfaces, we found that there are taxa that overlap between air and surface samples in both ISO-6–8.5 and ISO-5. In ISO-6–8.5 644 taxa are found in 90% or more of both Cat. 3 and Cat. 4 samples. For ISO-5 samples, 4 taxa are found in 90% or more of both Cat. 3 and Cat 4. samples.

### Samples include taxa with attributes useful to survive spaceflight

We then annotated the identified microbial species for the presence of planetary protection (PP) relevant properties, wherein they would be more likely to survive the rigors of spaceflight. Although there is no single set of properties that makes this possible, survival in space is generally associated with resistance to cold, radiation, and desiccation. These properties, in turn, are often found in microbes which can form spores, biofilms, or that are found in extreme locations on earth. We annotated observed microbes for these features using The Microbe Directory [34]. The Microbe Directory includes annotations for species known to be radiophilic, psychrophilic, found in extreme environments, and to form spores or biofilms. We expanded this list to note when a species belonged to a genus where one or more constituent species possessed these properties.

Though useful, The Microbe Directory is almost certainly an incomplete list of microbes with relevant PP properties. Thus, we sought to more fully annotate taxa by identifying whether a taxon or its close relatives were found in harsh environments, namely deserts, polar regions, or deep-water basins (based on data in the Earth Microbiome Project [45]). We performed a similar analysis for taxa in the MetaSUB dataset [40]: Identifying taxa found on stone/concrete, metal, or plastic surfaces in the urban environments.

**Fig. 3:**
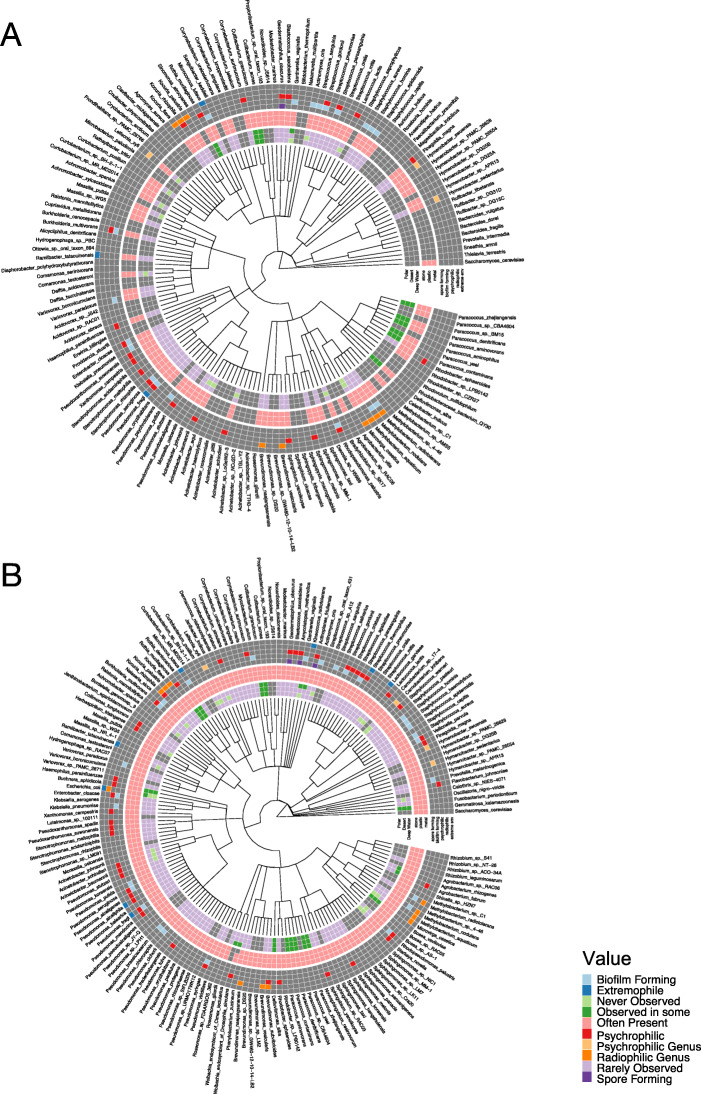
Microbial profiling. **a** Characteristics of all taxa detected in ISO-5 samples. **b** Characteristics of taxa in ISO-6–8.5 samples which were at 0.35% abundance in at least one sample

Microbial profiling shows the presence of biofilm-forming species from genera *Staphylococcus* and *Streptococcus*, and the spore-forming *Geodermatophilus obscurus* in both ISO groups (Fig. 3a, b). Multiple spore-forming bacteria were also annotated. Psychrophilic genera such as *Flavobacterium*, *Kocuria*, *Pseudomonas*, *Stenotrophomonas* were listed in both groups, as well as the radiophilic genera *Methylobacterium*, *Brevundimonas*, and *Kocuria*. Other annotated extremophiles were *Ramlibacter tataouinensis*, *Micrococcus luteus*, and *Streptococcus thermophilus*. Extremophiles *Pseudomonas alcaliphila* and *Pseudomonas fragi* were only found in samples from ISO-6–8.5. We also noted that most of the identified taxa were gram-negative species and were either strict aerobes or facultative anaerobes.

### Clean rooms contain novel taxa with genes associated with survival

We next computationally isolated several metagenome-assembled genomes (MAGs) from our samples to discover potentially new species. Of the 22 isolated, non-duplicate genomes, 12 did not match any known species. In all 12 cases, these genomes could be assigned to a genus and some genomes may have been multiple strains from the same previously unknown species. The detection of possible novel species suggests that clean rooms may select for specific adaptations, which is certainly plausible given the unique selection properties of the clean room environment. If the clean room environment continuously selects for high fitness species, it is plausible that new species will continuously emerge. This presents a difficulty for microbial characterization efforts, since novel species have not been previously characterized (by definition) but may present a planetary contamination risk.

**Fig. 4:**
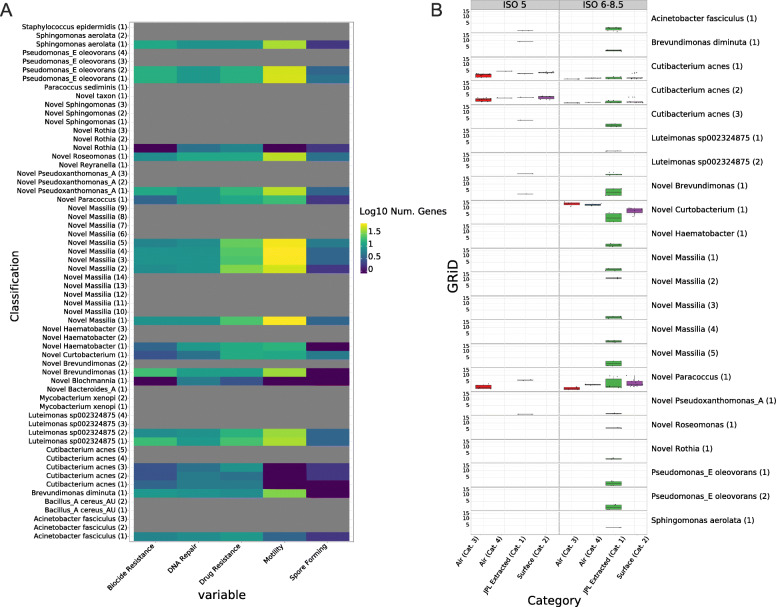
Novel genomes. **a** Number of likely novel bacterial species in the data. Novel species have necessarily not been characterized. To estimate the possible function of novel species we identify genes with known planetary protection significance in the genomes. **b** Growth rate (GRiD) scores for genomes computationally isolated. Numbers above 1 indicate active growth. Genomes that did not match any known species are referred as novel. Numbers next to species names indicate strains

To assess the risk presented by the novel genomes, we identified specific genes associated with strong-survivor characteristics. We grouped these genes into 5 categories: Biocide resistance (including resistance to cleaning agents), DNA repair (strongly related to radiation and desiccation resistance), drug resistance (antibiotics and similar), motility, and the ability to form spores. For each novel genome, we counted the number of genes in each category (Fig. 4) as a rough proxy for certain traits. We identified many of these genes associated with each trait among the different new genomes. However, we noted that both DNA repair and motility genes can be found in microbes that are not necessarily strong survivors, and genes for spore-forming do not always ensure that capacity.

### Microbes in clean rooms may be actively reproducing

We then estimated the rate of growth for major taxa using the Growth Rate Index (GRiD) [36]. GRiD uses the peak-to-trough ratio of coverage on a microbial genome and an iterative series of filters to estimate that genome’s rate of replication. In general, bacterial species replicate their genomes starting from a fixed origin of replication. At any given time, some fraction of a given bacterium in a sample will be at different stages of reproduction, which is represented as a higher copy number near the origin replication when actively dividing. By comparing the copy number at an origin of replication to the opposite total, the growth rate index may be estimated. GRiD is designed to work with low-coverage samples and low-quality genome assemblies. We filtered all GRiD estimates with strain heterogeneity above 0.5 as these estimates are likely to be inaccurate, for both, the reference strains, and the novel MAGs (Fig. 4). GRiD scores above 1 indicate that a bacteria is likely growing. We performed a one-sided t-test on all distributions of GRiD scores for each ISO level and category and removed all species which did not have GRiD distributions significantly higher than 1 in at least one category.

We noted a large number of possibly growing or persisting bacteria in the clean room samples. Generally, these were found in clean rooms with ISO-6–8.5 and mostly in the JPL extracted DNA (Cat. 1). The bacteria growing in all categories (Cat. 1 to Cat. 4) for ISO-6–8.5 corresponded to two strains of *Cutibacterium acnes*, and two novel *Curtobacterium* and *Paracoccus* strains. For ISO-5 clean rooms, fewer growing taxa were identified.

### Reproducibility testing

**Fig. 5:**
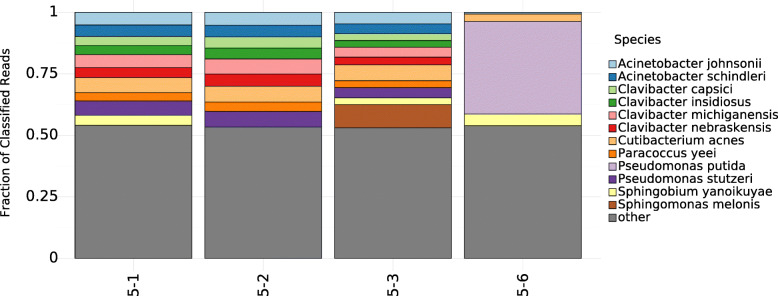
Method reproducibility. Taxonomic profiles of multiple replicates of the same sample

In order to establish our technical methods as valid, we assessed our methods ability to produce reproducible results. The same test was performed on the same sample (multiple replicates were collected) five times to account for possible variations. Of the replicates we identified very similar taxonomic profiles for three of the replicates and one divergent taxonomic profile (Fig. 5). One replicate had no identifiable species after filtering and was excluded from further analysis.

## Discussion

Planetary protection efforts endeavor to preserve extraterrestrial bodies for future scientific investigation and to minimize the risk of exposure to contamination materials from outer space missions [12]. Clean rooms at the Jet Propulsion Laboratory at NASA are kept with the highest standards of cleanliness using filtered air circulation, controlled temperature and humidity, routine exposure to disinfectants, and surfaces cleaning [46, 47]. The certification of cleanrooms is described by the number of particles in the air. The certification standards classified by the International Organization for Standardization (ISO) sets these requirements and characteristics for cleanroom operations [48, 49]. ISO classes represent the maximum number of particles greater than 0.5μm per cubic meter. Spacecraft assembly cleanrooms range from ISO-4 to ISO-8. In addition, air is filtered with high-efficiency particulate air (HEPA) or ultra-low penetration air (ULTA) filters that remove 99.97% or 99.99% of particulates ≥ 0.3 μm, respectively. The temperature, humidity, and pressure are also controlled [3]. These severe standards in clean rooms at the Spacecraft Assembly Facilities may act as a selective factor for microorganisms that can tolerate and thrive in oligotrophic conditions, high UV exposures, and cleaning agents [6].

Here, we presented a standardized metagenomic processing workflow and analysis pipeline for spaceflight hardware cleanroom samples to identify microbial contamination, even at low abundances. We described the potential risk of contamination and their viability to grow in such conditions based on the ecological characteristics of these species. We were able to identify samples of microbial composition, with phyla Actinobacteria, Proteobacteria, and Firmicutes to be among the most abundant. These analyses showed that samples from ISO-6–8.5 were the most diverse and had the higher number of reads from the microbes present. Although there were common microorganisms present in ISO-6–8.5 and ISO-5, the latter had significantly less richness and diversity. When comparing the number of microorganisms found in filters and surfaces in both ISO, we found 644 common species in ISO-6–8.5 and only 4 in ISO-5. These results can be explained by the number of particles that each cleanroom standard allows per volume of air: The higher the standard, the bigger the particle size permitted per cubic foot of air [50].

Microbial profiling showed that taxa from ISO-6–8.5 are often found in materials such as metal, plastic, and stone, yet not all taxa in ISO-5 are found in such materials. However, the microbial features annotated on the microbial profiling revealed the presence of planetary protection relevant microbes in both ISO-5 and ISO-6–8.5 such as the extremophile *Ramlibacter tataouinensis*, first isolated from a meteorite fragment in the Tataouine desert in Tunisia [51]. This desiccation-tolerant bacteria belongs to the class Betaproteobacteria and has among the highest G+C content (70%) in the class, notably presenting with two cell shapes (spherical and rod) that are thought to help with adaptation to desert environments [52] (cycles of air-drying, rehydration, and long-term desiccation). The species *Geodermatophilus obscurus*, a member of the phylum Actinobacteria, was also found in both ISO-5 and ISO-6–8.5 and has also been isolated from desert environments. It is also known to be UVC and gamma-ray resistant [53, 54]. Other extremophile genera such as *Flavobacterium*, *Kocuria*, *Pseudomonas*, *Stenotrophomonas*, *Micrococcus*, and *Paracoccus* have been reported in previous studies in clean rooms [6, 7, 55]. Interestingly, the genus *Kocuria* has been shown to be capable to grow in Mars-like soil conditions with high levels of perchlorates [56], which might represent a potential risk for Martian missions.

When trying to assess the risk presented by the microorganisms, we identified specific genes associated with strong-survivor characteristics based on reference databases. However, we noted that both DNA repair and motility genes were also found in microbes that are not strong survivors. These results evidence that more robust bioinformatic or culture-dependent analysis must be performed to assess a particular microbe resistance to radiation, desiccation, or other forces present in spaceflight. As more samples are taken from the facilities in this study as part of longitudinal series, it will be possible to evaluate selective pressure on individual genes and organisms. This approach has been applied by Sghaier et al. [57] to identify positive selection on DNA repair genes in radiation-resistant organisms in silico.

Although the extensive cleaning processes limit the number of microbes capable of withstand such clean room conditions, hardy spore-forming microorganisms like *Amycolatopsis methanolica* (also a methylotrophic bacteria) [58], *Actinoplanes friuliensis*, and *Geodermatophilus obscurus*, demonstrate the capacity of certain taxa to survive sterilization processes. However, it is important to identify and assembly microorganisms that may be actively growing [59]. To measure the microbial growth rate we used Growth Rate Index (GRiD), a bioinformatic tool able to work with low-quality genome assemblies even at 0.05% relative abundances of 100 bp × 10 million reads. Actively growing bacteria were found in all categories (Cat. 1 to Cat. 4). For ISO-6–8.5 cleanroom standards, these bacteria corresponded to two strains of *Cutibacterium acnes*, and two novel *Curtobacterium* and *Paracoccus* strains. While for ISO-5 only few growing taxa were identified.

To keep record of the microbes that persist in JPL clean rooms, other SAFs, and to strengthen cleaning protocols, reproducibility and biological threshold are needed. These data provide a useful guide to such metrics. We establish our technical methods and pipelines as valid supported by the reproducibility analysis. Although there was one replicate that had no identifiable species after filtering and another with a divergent taxonomic profile (likely due to contamination by *Pseudomonas putida*), results showed very similar taxonomic profiles in three out of four replicated samples, discarding any biological misinterpretation due to technical error. Altogether, our comprehensive metagenomics framework gives insights into the microbes present in clean rooms, which might represent a risk for planetary missions based on their genetic and phenotypic traits.

## Conclusions

Although the extensive cleaning processes limit the number of microbes capable of withstanding clean room condition, it is important to quantify thresholds and detect organisms that can inform ongoing Planetary Protection goals, provide a biological baseline for assembly facilities, and guide future mission planning.

## Supplementary Information

**Additional file 1: Table S1.** Sample Sets and Categories for JPL Planetary Protection Testing

**Additional file 2: Figure S1.** Distribution of Species Abundances The distribution of the maximum observed relative abundance of each species in a single sample for ISO-5 samples (**A**) and ISO-6-8.5 samples (**B**).

**Additional file 3: Figure S2.** Distribution of QC Metrics. The distributions of various QC metrics for controls and ISO levels. Distributions of QC metrics for non-control samples are largely unaffected by category which suggests good overall quality. **A**) Distribution of read count. **B**) Coverage of marker k-mer set for each taxa detected in samples. **C**) Number of unique marker k-mers for each taxa detected in samples. **D**) The ratio of unique marker k-mers to read count for each taxa detected in sample. A low ratio (about 1) could indicate a false positive.

**Additional file 4: Figure S3.** Comparison of QC Metrics in cases to controls. We detected 24 species across all control samples after initial filtering. Environmental metagenomic samples are qualitatively similar to samples of laboratory contamination so filtering based on controls is non-trivial. We compared the number of unique marker k-mers found for each taxa in each case sample to the maximum number found in one control (**A**) and, analogously, the total number of reads (**B**). We took all taxonomic assignments with a read ratio greater than 2 and a k-mer ratio greater than 5 (**C**). If a taxonomic assignment met both criteria in at least two samples, it was permitted (red taxa on bottom right) otherwise the taxa was filtered from all assignments (blue taxa on bottom right).

**Additional file 5: Figure S4.** Relationship of QC Values to Taxonomic Assignment. **A.** Total number of reads per sample, note log scale on y-axis. **B.** Top-Left) Diversity of marker k-mers compared to diversity of identified taxa measured by Shannon’s entropy, Pearson’s correlation coefficients rho=0.625. Top-Right) Fraction of singleton k-mers compared to diversity of identified taxa, rho=0.499. Bottom-Right) Read count compared to diversity of identified taxa, rho=0.781. Bottom-Left) Read count compared to total number of detected species (rarefied to 1,000 reads), rho=0.886.

**Additional file 6: Figure S5.** Rarefaction Analysis. The number of unique species detected in multiple samples of the same type. **A**) Sample sets appear to reach a maximum suggesting that the majority of species have been fully categorized in each category and ISO level. **B**) number of species detected when individual samples are rarefied to a maximum read count

**Additional file 7: Figure S6.** Effect of QC Parameters on Taxa Richness. Various QC parameters have an effect on the number of species detected. These plots show the effect of marker-set coverage (color), minimum read count (horizontal panels numbered 1, 2, 3), and minimum number of unique marker k-mers (x-axis of each panel). The y-axis of each panel shows the number of taxa passing the requisite criteria for genus (top panels) and species (bottom panels.) **A**) ISO-5 samples **B**) ISO-6-8.5 samples.

**Additional file 8: Figure S7.** Prevalence of species in different categories. Heatmap showing the prevalence (fraction of samples where a taxon is detected) of different taxa across categories. Too many taxa are present to individually name taxa.

## Data Availability

Raw sequencing data, results, and visualizations can be accessed at https://pngb.io/jpl-clean-rooms. Taxonomic profiles and other generated analyses are available in the supplement of this study. All figures presented in this manuscript may be generated using code also available in the supplement. The bulk of initial analysis was performed using the publicly available MetaSUB Core Analysis Pipeline https://github. com/MetaSUB/MetaSUB_CAP.
