# Supplementary material for: A comprehensive metagenomics framework to characterize organisms relevant for planetary protection
Source: Microbiome. 2021 Apr 1;9:82. doi: 10.1186/s40168-021-01020-1 (PMC8016160; doi:10.1186/s40168-021-01020-1)
Supplement: Supplementary file 2 — Additional file 1: Table S1. Sample Sets and Categories for JPL Planetary Protection Testing [file 40168_2021_1020_MOESM2_ESM.pdf]

Sample Name	Sample Type	Sample Category	Sample Description	Total Area Sampled	Amount Shipped	Date Shipped to Vendors	Flowcell ID	Barcode	# of Reads	%>Q30	Mean Q score
Buffer Control	Control	Control	Used for sample dissociation	N/A	10ul	4/15/19	Buffer_Control	AACACTGTTA+TGAGACTTGC	67,262	81.11	35.28
Elution Buffer	Control	Control	Used for DNA elution	N/A	10ul	4/15/19	Elution_Buffer	GTCCGTAAGC+AAGATACACG	1,784	68.19	31.9
Sample 1-1	DNA	1	ISO 7 (170-213) Facility	17.5m^2	10ul	4/15/19	1_1	ACTTCAAGCG+TTCATGGTTC	2,945	72.64	33.02
Sample 1-2	DNA	1	Facility Control	N/A	10ul	4/15/19	1_2	TCAGAAGGCG+TATGATGGCC	902	60.75	29.94
Sample 1-3	DNA	1	ISO 5 (233-151) Facility	22m^2	10ul	4/15/19	1_3	GCGTTGGTAT+GGAAGTATGT	1,911	69.16	32.07
Sample 1-4	DNA	1	Extraction Control	N/A	10ul	4/15/19	1_4	ACATATCCAG+ATTGCACATA	4,700	76.5	34.04
Sample 1-5	DNA	1	ISO 5 (233-151) Filter	N/A	10ul	4/15/19	1_5	TCATAGATTG+CACCTTAATC	1,912	70.9	32.59
Sample 1-6	DNA	1	Filter control	N/A	10ul	4/15/19	1_6	GTATTCCACC+TTGTCTACAT	3,868	75.43	33.77
Sample 1-7	DNA	1	ISO 5 (233-151) Facility	27 m^2	10ul	4/15/19	1_7	GTCCGTAAGC+AAGATACACG	9,916,177	80.05	35.01
Sample 1-8	DNA	1	ISO 7 (179-121) Filter	N/A	10ul	5/6/19	1_8	TGATGTAAGA+AAGGACGCAC	23,586,161	80.71	35.15
Sample 1-9	DNA	1	ISO 8.5 (103-110) Facility	5m^2	10ul	5/6/19	1_9	GGAATTGTAA+AGGATGTGCT	5,338	80.01	34.96
Sample 1-10	DNA	1	ISO 8.5 (103-110) Facility	4m^2	10ul	5/6/19	1_10	GCATAAGCTT+TGCGACGGAA	15,371,175	80.58	35.11
Sample 1-11	DNA	1	ISO 6 (103-102C) Facility	3m^2	10ul	5/6/19	1_11	CCTCCGTCCA+CACCGATGTG	489,203	83.11	35.8
Sample 1-12	DNA	1	ISO 5 (233-151) Facility	17m^2	10ul	5/6/19	1_12	CCTCCGTCCA+CACCGATGTG	16,082	82.02	35.49
Sample 2-1	Wipe Solution	2	ISO 7 (170-213) Facility	17.5m^2	40ml	4/15/19	2_1	CTCGCTTCGG+TTGACTAGTA	4,838,770	77.75	34.35
Sample 2-2	Wipe Solution	2	ISO 5 (233-151) Facility	22m^2	40ml	4/15/19	2_2	TCTATCCTAA+CCAGTCGACG	141,971	86.91	36.81
Sample 2-3	Wipe Solution	2	Facility Control	N/A	40ml	4/15/19	2_3	CTGTTGGTCC+AACGGTCTAT	417,514	80.53	35.09
Sample 2-4	Wipe Solution	2	ISO 5 (233-151) Facility	27 m^2	40ml	4/15/19	2_4	TTACCTGGAA+CTGGAACTGT	178,792	81.26	35.3
Sample 2-5	Wipe Solution	2	ISO 6 (103-102C) Facility	3m^2	40ml	4/15/19	2_5	TGGCGCGAAC+AGTATCAGTT	22,972,146	76.5	34.01
Sample 2-6	Wipe Solution	2	ISO 8.5 (103-110) Facility	5m^2	40ml	5/6/19	2_6	TAATGTGTCT+TATGCCTTAC	8,671,128	81.5	35.36
Sample 2-7	Wipe Solution	2	ISO 8.5 (103-110) Facility	4m^2	40ml	5/6/19	2_7	ATACCAACGC+CGCAGCAATT	24,558,417	81	35.22
Sample 2-8	Wipe Solution	2	ISO 8.5 (103-110) Facility	4m^2	40ml	5/6/19	2_8	AGGATGTGCT+GGAATTGTAA	8,251,741	78.69	34.61
Sample 2-9	Wipe Solution	2	ISO 8.5 (103-110) Facility	4m^2	40ml	5/6/19	2_9	CACGGAACAA+GTGCTAGGTT	12,412,207	75.19	33.64
Sample 2-10	Wipe Solution	2	ISO 5 (233-151) Facility	17m^2	40ml	5/6/19	2_10	TGGAGTACTT+TCCACACAGA	67,143	82.43	35.6
Sample 2-11	Wipe Solution	2	ISO 6 (103-102C) Facility	3m^2	40ml	5/6/19	2_11	GTATTGACGT+TTGGAATTCC	QC Fail	0	0
Sample 2-12 (1)	Control	2	Extraction Control - neg1	N/A	10uL	*on site*	2_12	AGTGTTGCAC+CGTGTACCAG	2	56.83	28.91
Sample 2-12 (2)	Control	2	Extraction Control - neg2	N/A	10uL	*on site*	2_12	GACACCATGT+TACACGTTGA	1	88	37.07
Sample 2-12 (3)	Control	2	Extraction Control - neg3	N/A	10uL	*on site*	2_12	CCTGTCTGTC+TCACAACAGT	5	78.47	34.78
Sample 3-1	Filter Solution	3	ISO 5 (233-151) Carbon Filter	N/A	20ml	4/15/19	3_1	TGGCTAATCA+CTACATGCCT	148,906	83.01	35.76
Sample 3-2	Filter Solution	3	Filter control	N/A	20ml	4/15/19	3_2	ATTGCGCGGT+GCGGAGCCAA	49,277	81.11	35.23
Sample 3-3	Filter Solution	3	ISO 5 (233-151) Carbon Filter	N/A	30ml	5/6/19	3_3	CTTGTACACC+AAGCGCGCTT	1,541,796	81.26	35.28
Sample 3-4	Filter Solution	3	ISO 5 (233-151) Carbon Filter	N/A	30ml	5/6/19	3_4	ACACAGGTGG+ACAACGCTCA	174,614	81.52	35.34
Sample 3-5	Filter Solution	3	ISO 5 (233-151) Carbon Filter	N/A	30ml	5/6/19	3_5	CCTGCGGAAC+AGCCTATGAT	188,576	80.37	35.04
Sample 3-6	Filter Solution	3	ISO 5 (233-151) Carbon Filter	N/A	30ml	5/6/19	3_6	TTCATAAGGT+CCTTCTAACA	639,408	81.32	35.31
Sample 3-7	Filter Solution	3	ISO 8 (318-123) non-carbon filter	N/A	30ml	5/6/19	3_7	CTCTGCAGCG+TACATCCATC	46,952,590	79.68	34.86
Sample 3-8	Filter Solution	3	ISO 7 (306) non-carbon filter	N/A	30ml	5/6/19	3_8	CTGACTCTAC+TGACGGCCGT	8,240,058	83.05	35.73
Sample 3-9	Filter Solution	3	ISO 5 (233-151) Carbon Filter	N/A	30ml	5/6/19	3_9	TCTGGTATCC+GTAAGCAACG	214,218	81.8	35.41
Sample 3-10	Filter Solution	3	Non-carbon filter control	N/A	30ml	5/6/19	3_10	CATTAGTGCG+TATGTAGTCA	91,903	81.32	35.29
Sample 4-1	Vacuum Particle Solution	4	ISO 7 (179-121) Vacuum #1	N/A	30ml	5/6/19	4_1	ACGGTCAGGA+AACGAGGCCG	23,247,564	76.58	34.05
Sample 4-2	Vacuum Particle Solution	4	ISO 7 (179-121) Vacuum #2	N/A	30ml	5/6/19	4_2	GGCAAGCCAG+CGGATGCTTG	19,061,448	76.37	33.99
Sample 4-3	Vacuum Particle Solution	4	ISO 7 (179-121) Vacuum #3	N/A	30ml	5/6/19	4_3	TGTCGCTGGT+AGTCAGACGA	25,126,057	79.27	34.76
Sample 4-4	Vacuum Particle Solution	4	ISO 7 (179-121) Vacuum #4	N/A	30ml	5/6/19	4_4	ACCGTTACAA+TCGCTATGAG	26,721,229	75.53	33.78
Sample 4-5	Vacuum Particle Solution	4	ISO 7 (179-121) Vacuum #5	N/A	30ml	5/6/19	4_5	TATGCCTTAC+TAATGTGTCT	23,528,796	78.88	34.66
Sample 4-6	Vacuum Particle Solution	4	ISO 5 (233-141) Vacuum #1	N/A	30ml	5/6/19	4_6	ACAAGTGGAC+AACATCGCGC	1,436,089	80.76	35.15
Sample 4-7	Vacuum Particle Solution	4	ISO 5 (233-141) Vacuum #2	N/A	30ml	5/6/19	4_7	TGGTACCTAA+AGTACTCATG	1,219,598	80.63	35.13
Sample 4-8	Vacuum Particle Solution	4	ISO 5 (233-141) Vacuum #3	N/A	30ml	5/6/19	4_8	TTGGAATTCC+GTATTGACGT	2,424,418	80.03	34.95
Sample 4-9	Vacuum Particle Solution	4	ISO 5 (233-141) Vacuum #4	N/A	30ml	5/6/19	4_9	CCTCTACATG+AGGAGGTATC	648,851	79.92	34.93
Sample 4-10	Vacuum Particle Solution	4	ISO 5 (233-141) Vacuum #5	N/A	30ml	5/6/19	4_10	GGAGCGTGTA+ACTTACGGAT	2,500,687	79.33	34.77
Sample 5-1	DNA	5	SAF DNA replicates	N/A	10ul	4/15/19	5_1	ACTTCAAGCG+TTCATGGTTC	36,261,784	75.86	33.9
Sample 5-2	DNA	5	SAF DNA replicates	N/A	10ul	4/15/19	5_2	TCAGAAGGCG+TATGATGGCC	38,989,874	74.37	33.52
Sample 5-3	DNA	5	SAF DNA replicates	N/A	10ul	4/15/19	5_3	GCGTTGGTAT+GGAAGTATGT	8,248,701	82.27	35.56
Sample 5-4	DNA	5	SAF DNA replicates	N/A	10ul	4/15/19	5_4	ACATATCCAG+ATTGCACATA	QC Fail	0	0
Sample 5-5	DNA	5	SAF DNA replicates	N/A	10ul	4/15/19	5_5	TCATAGATTG+CACCTTAATC	5,157	75.44	33.8
Sample 5-6	DNA	5	SAF DNA replicates	N/A	10ul	4/15/19	5_6	GTATTCCACC+TTGTCTACAT	196,173,850	78.96	34.73
