# Supplementary figures and images for: A comprehensive metagenomics framework to characterize organisms relevant for planetary protection
Source: Microbiome. 2021 Apr 1;9:82. doi: 10.1186/s40168-021-01020-1 (PMC8016160; doi:10.1186/s40168-021-01020-1)

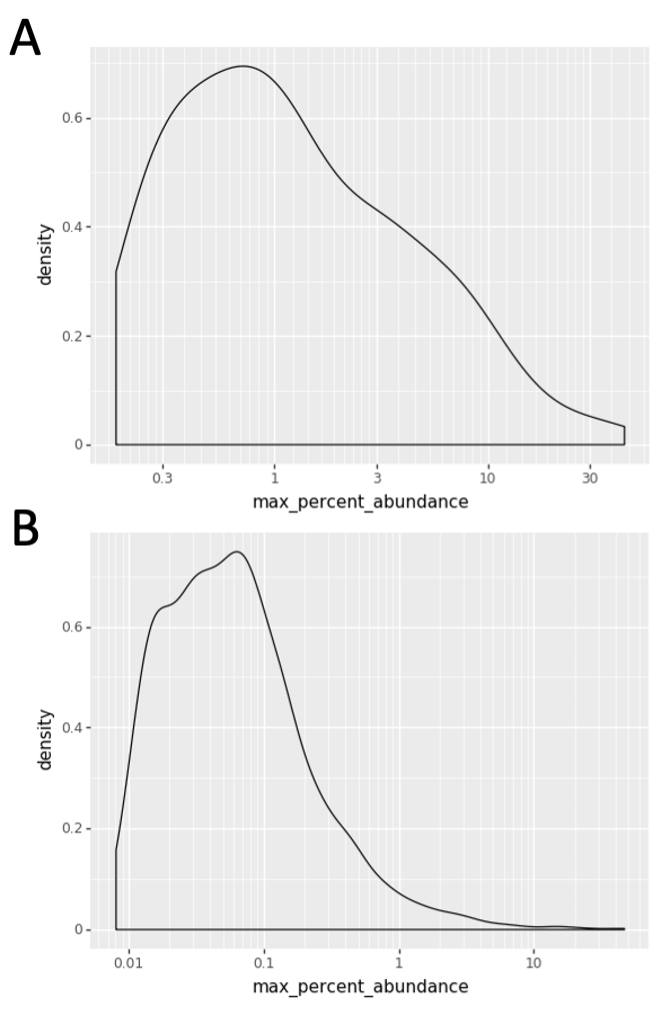

Supplement: Supplementary file 3 — Additional file 2: Figure S1. Distribution of Species Abundances The distribution of the maximum observed relative abundance of each species in a single sample for ISO-5 samples (A) and ISO-6-8.5 samples (B). [file 40168_2021_1020_MOESM3_ESM.png]

A

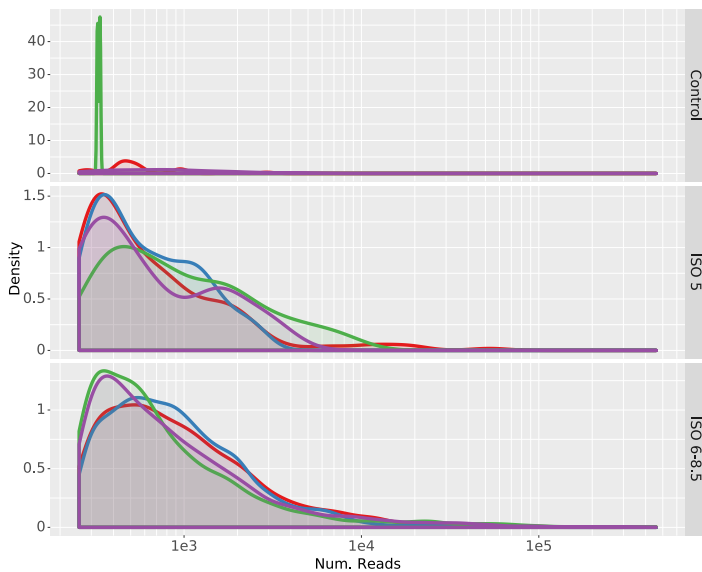

B

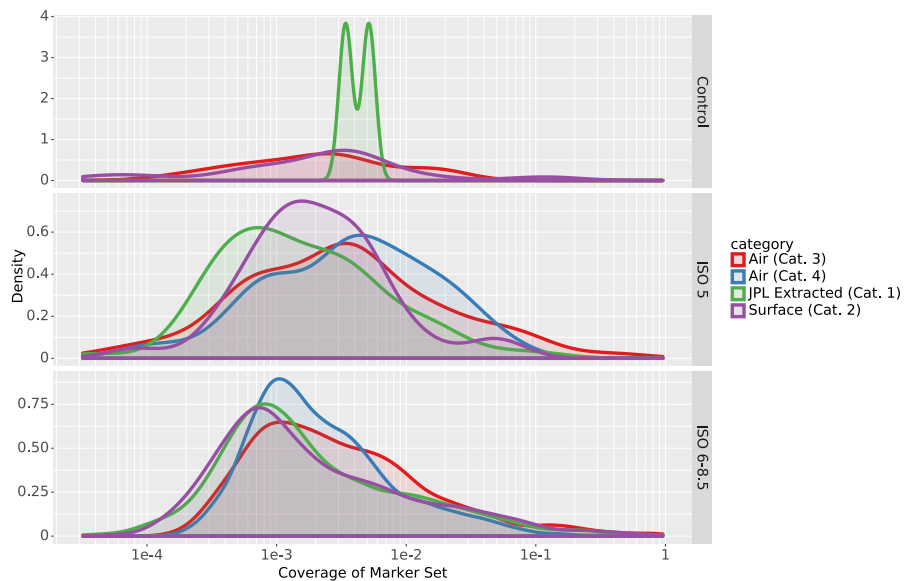

C

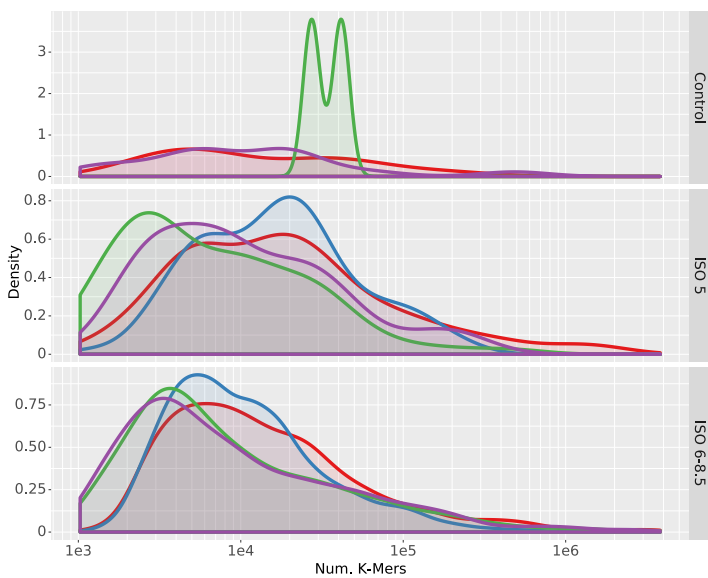

D

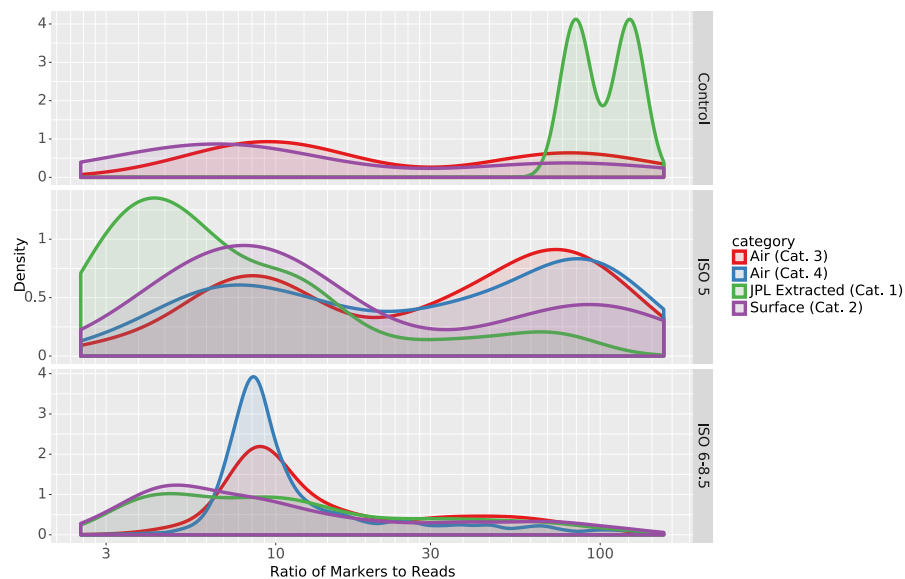

Supplement: Supplementary file 4 — Additional file 3: Figure S2. Distribution of QC Metrics. The distributions of various QC metrics for controls and ISO levels. Distributions of QC metrics for non-control samples are largely unaffected by category which suggests good overall quality. A) Distribution of read count. B) Coverage of marker k-mer set for each taxa detected in samples. C) Number of unique marker k-mers for each taxa detected in samples. D) The ratio of unique marker k-mers to read count for each taxa detected in sample. A low ratio (about 1) could indicate a false positive. [file 40168_2021_1020_MOESM4_ESM.pdf]

A

Ratio of unique k-mers in cases to controls

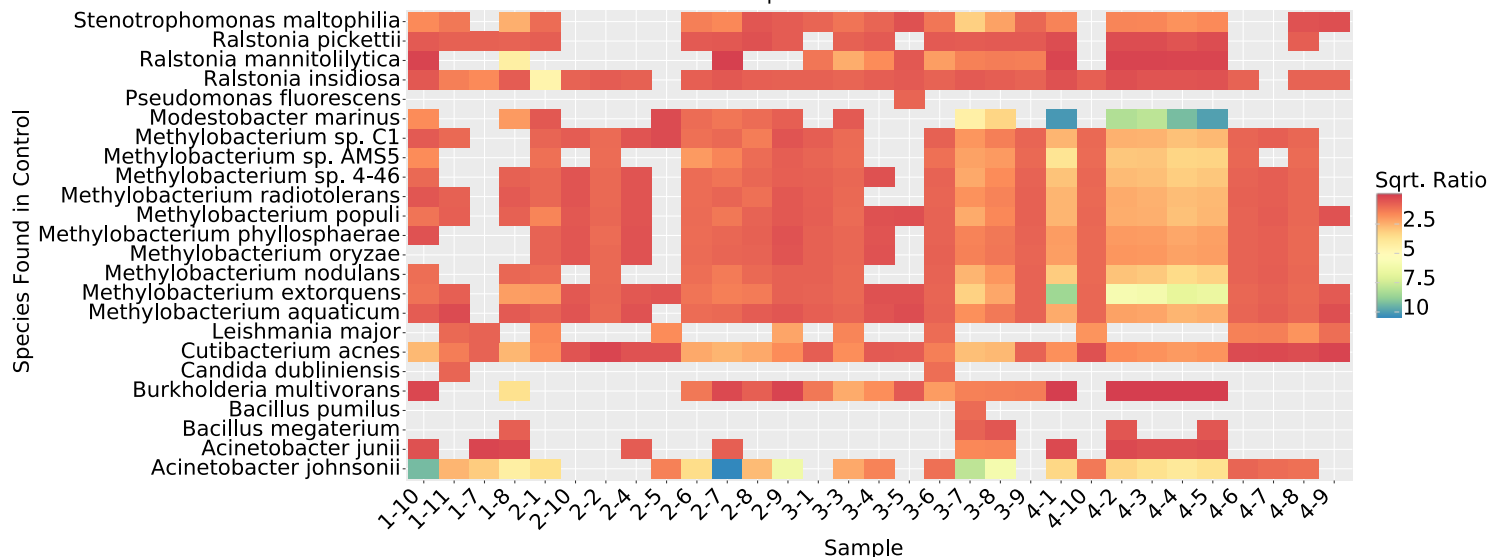

B

Ratio of total reads in cases to controls

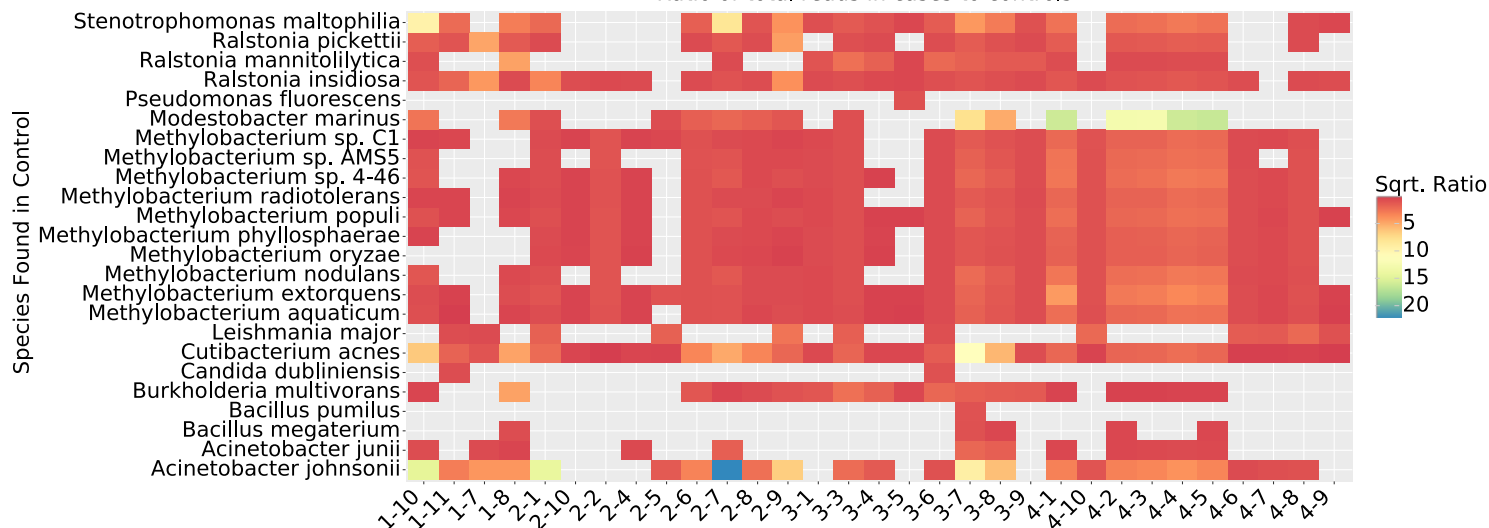

C

Mask

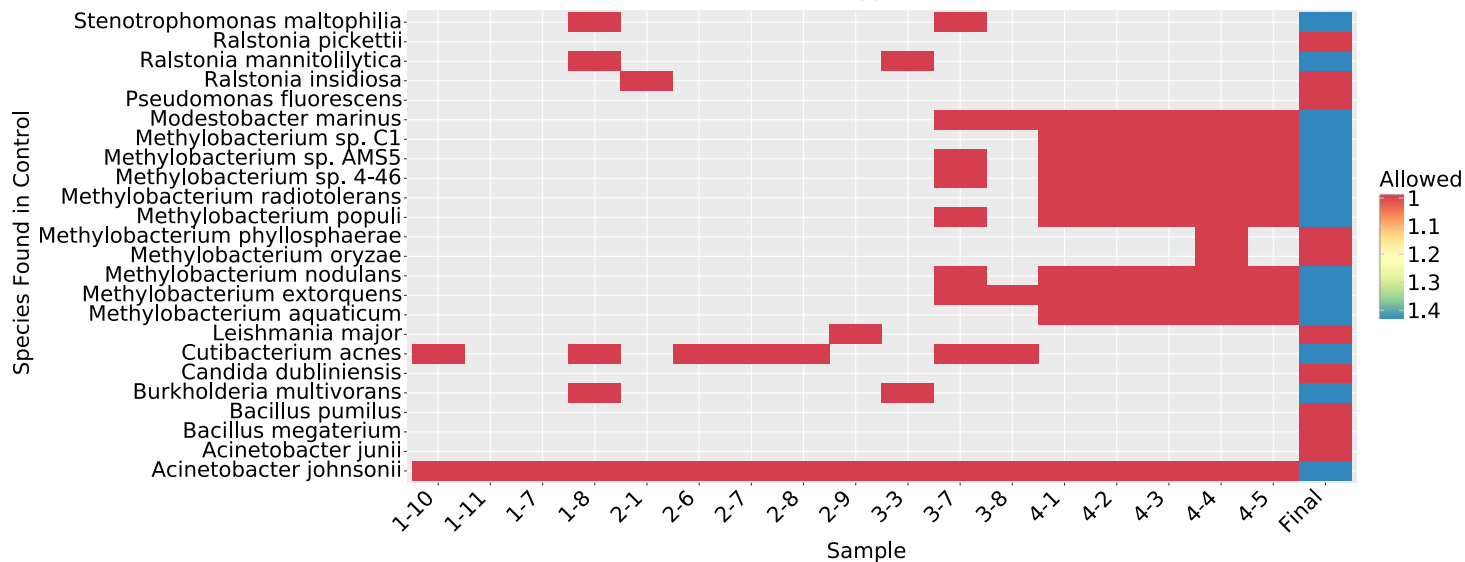

Supplement: Supplementary file 5 — Additional file 4: Figure S3. Comparison of QC Metrics in cases to controls. We detected 24 species across all control samples after initial filtering. Environmental metagenomic samples are qualitatively similar to samples of laboratory contamination so filtering based on controls is non-trivial. We compared the number of unique marker k-mers found for each taxa in each case sample to the maximum number found in one control (A) and, analogously, the total number of reads (B). We took all taxonomic assignments with a read ratio greater than 2 and a k-mer ratio greater than 5 (C). If a taxonomic assignment met both criteria in at least two samples, it was permitted (red taxa on bottom right) otherwise the taxa was filtered from all assignments (blue taxa on bottom right). [file 40168_2021_1020_MOESM5_ESM.pdf]

A

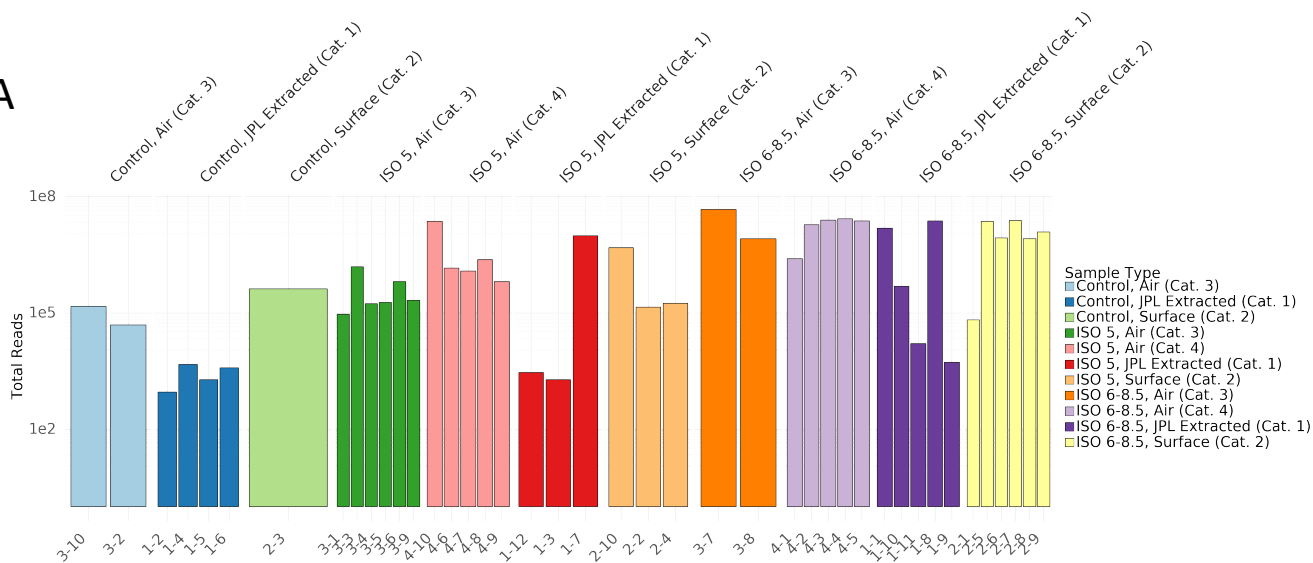

B

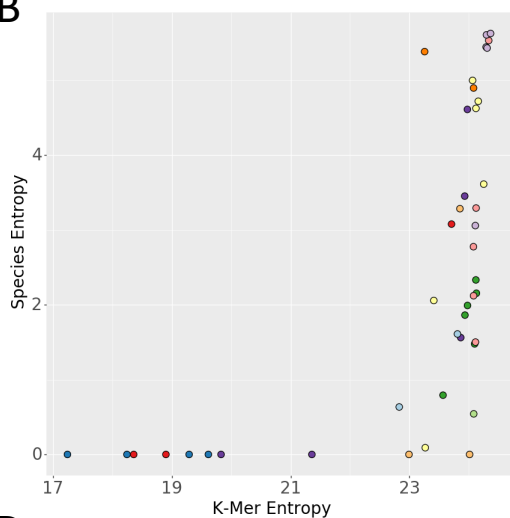

C

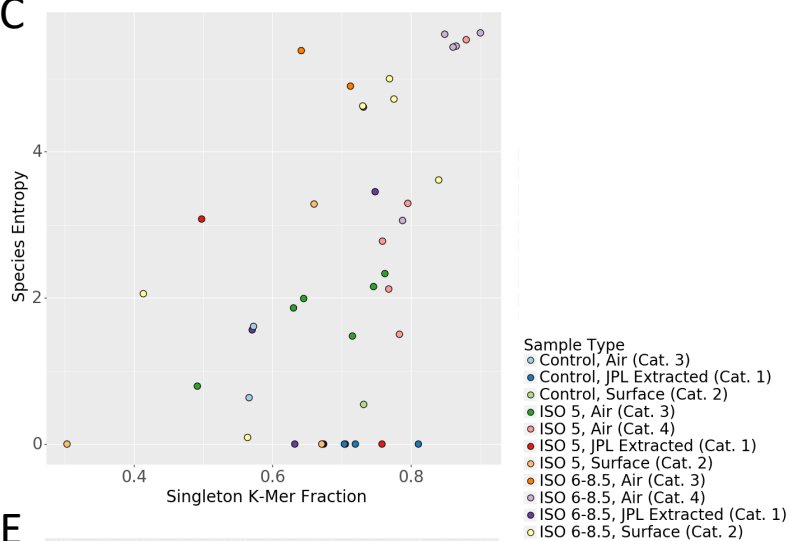

D

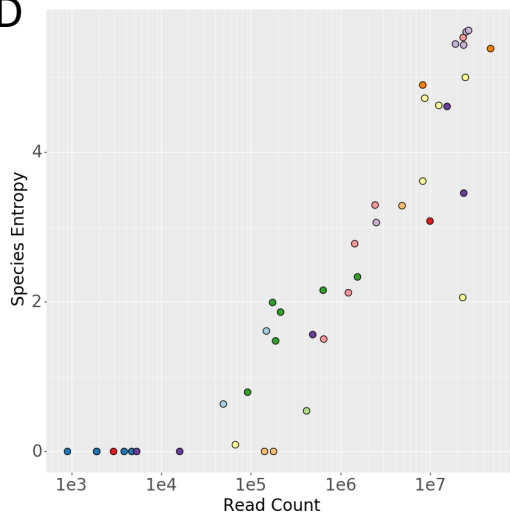

E

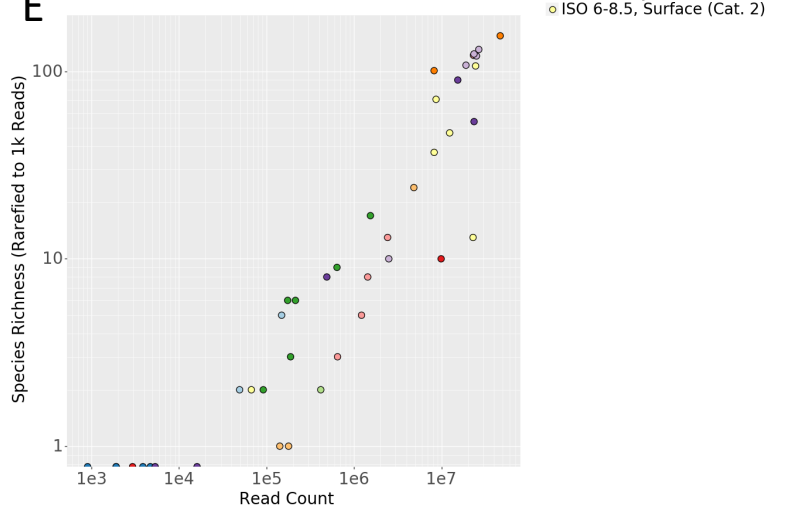

Supplement: Supplementary file 6 — Additional file 5: Figure S4. Relationship of QC Values to Taxonomic Assignment. A. Total number of reads per sample, note log scale on y-axis. B. Top-Left) Diversity of marker k-mers compared to diversity of identified taxa measured by Shannon’s entropy, Pearson’s correlation coefficients rho=0.625. Top-Right) Fraction of singleton k-mers compared to diversity of identified taxa, rho=0.499. Bottom-Right) Read count compared to diversity of identified taxa, rho=0.781. Bottom-Left) Read count compared to total number of detected species (rarefied to 1,000 reads), rho=0.886. [file 40168_2021_1020_MOESM6_ESM.pdf]

A

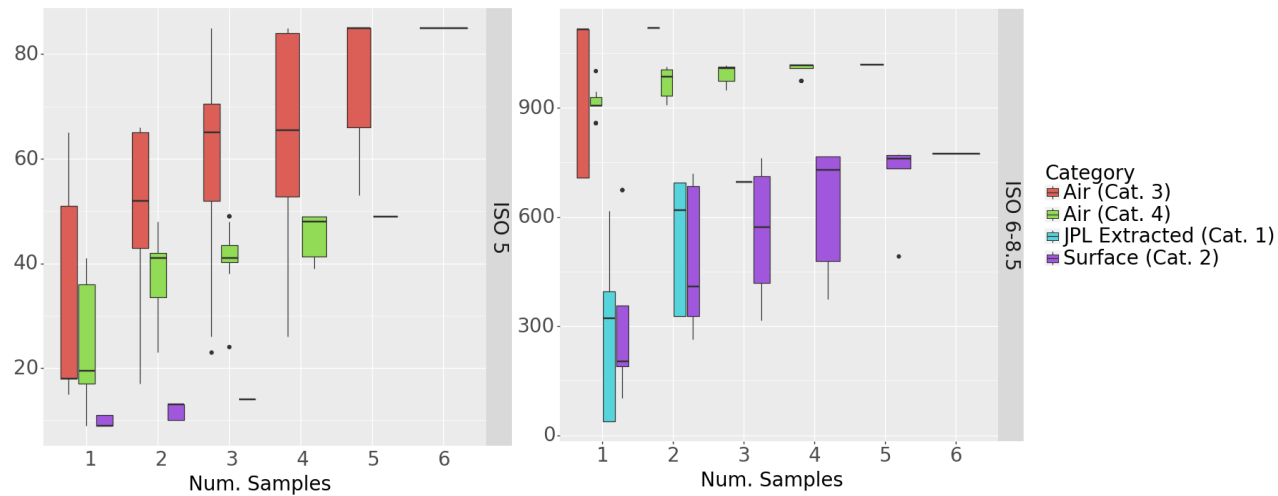

B

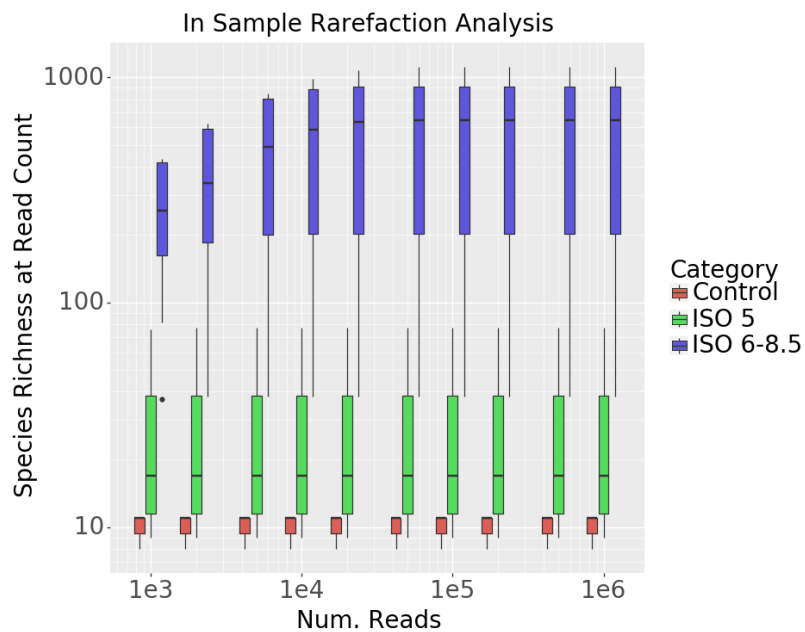

Supplement: Supplementary file 7 — Additional file 6: Figure S5. Rarefaction Analysis. The number of unique species detected in multiple samples of the same type. A) Sample sets appear to reach a maximum suggesting that the majority of species have been fully categorized in each category and ISO level. B) number of species detected when individual samples are rarefied to a maximum read count [file 40168_2021_1020_MOESM7_ESM.pdf]

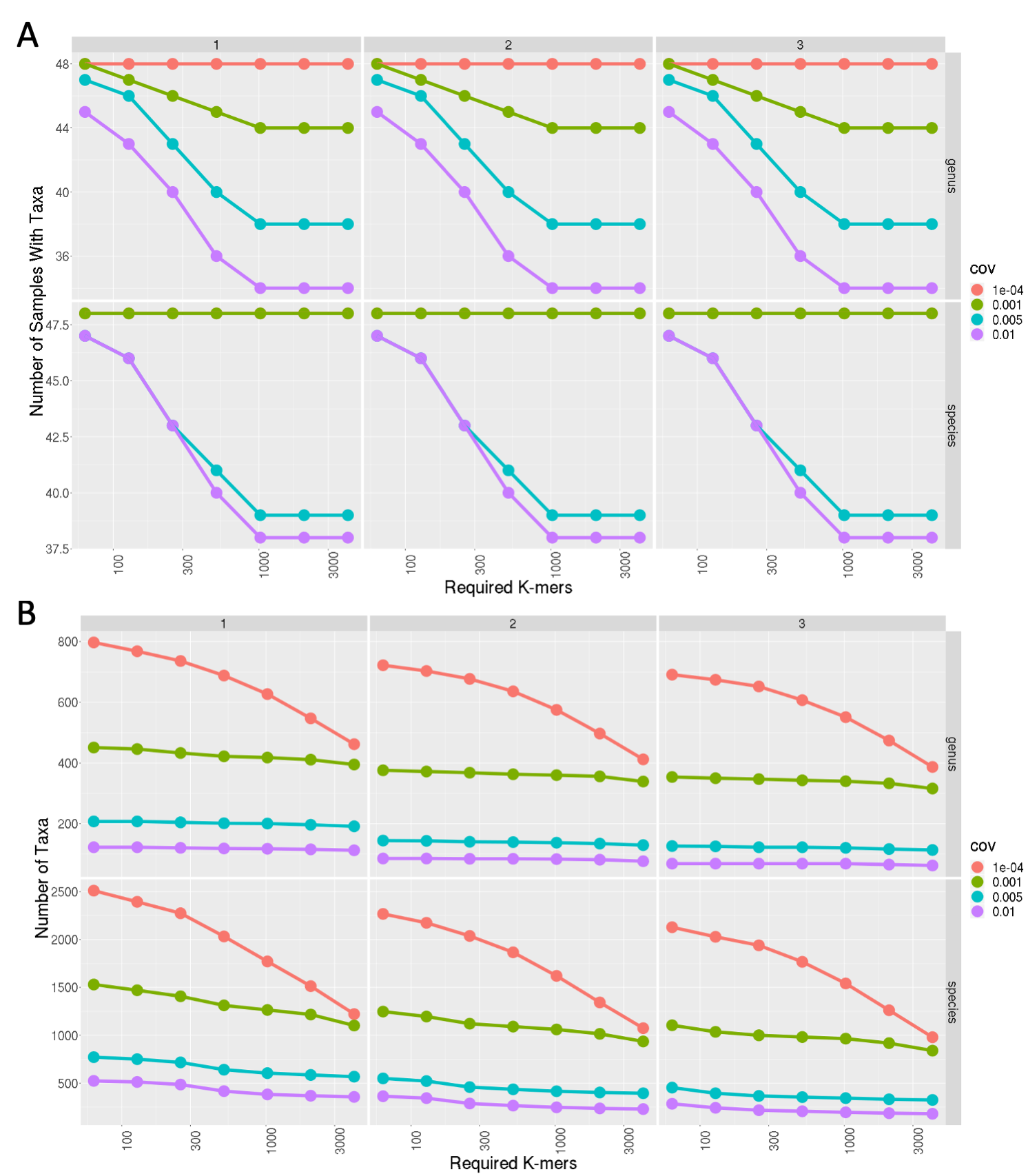

Supplement: Supplementary file 8 — Additional file 7: Figure S6. Effect of QC Parameters on Taxa Richness. Various QC parameters have an effect on the number of species detected. These plots show the effect of marker-set coverage (color), minimum read count (horizontal panels numbered 1, 2, 3), and minimum number of unique marker k-mers (x-axis of each panel). The y-axis of each panel shows the number of taxa passing the requisite criteria for genus (top panels) and species (bottom panels.) A) ISO-5 samples B) ISO-6-8.5 samples. [file 40168_2021_1020_MOESM8_ESM.png]

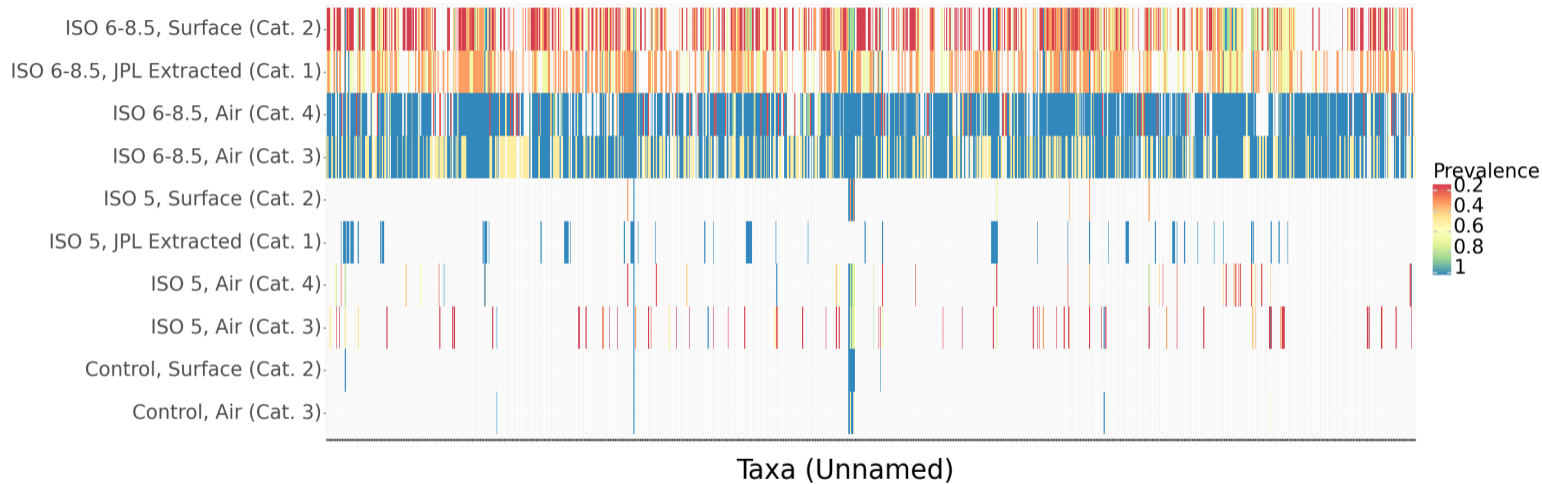

Supplement: Supplementary file 9 — Additional file 8: Figure S7. Prevalence of species in different categories. Heatmap showing the prevalence (fraction of samples where a taxon is detected) of different taxa across categories. Too many taxa are present to individually name taxa. [file 40168_2021_1020_MOESM9_ESM.pdf]
